# *Propionibacterium acnes* CAMP Factor and Host Acid Sphingomyelinase Contribute to Bacterial Virulence: Potential Targets for Inflammatory Acne Treatment

**DOI:** 10.1371/journal.pone.0014797

**Published:** 2011-04-12

**Authors:** Teruaki Nakatsuji, De-chu C. Tang, Liangfang Zhang, Richard L. Gallo, Chun-Ming Huang

**Affiliations:** 1 Division of Dermatology, Department of Medicine, University of California San Diego, San Diego, California, United States of America; 2 VA San Diego Healthcare Center, San Diego, California, United States of America; 3 Vaxin Inc., Birmingham, Alabama, United States of America; 4 Department of Nanoengineering, University of California San Diego, San Diego, California, United States of America; University of Liverpool, United Kingdom

## Abstract

**Background:**

In the progression of acne vulgaris, the disruption of follicular epithelia by an over-growth of *Propionibacterium acnes* (*P. acnes*) permits the bacteria to spread and become in contact with various skin and immune cells.

**Methodology/Principal Findings:**

We have demonstrated in the present study that the Christie, Atkins, Munch-Peterson (CAMP) factor of *P. acnes* is a secretory protein with co-hemolytic activity with sphingomyelinase that can confer cytotoxicity to HaCaT keratinocytes and RAW264.7 macrophages. The CAMP factor from bacteria and acid sphingomyelinase (ASMase) from the host cells were simultaneously present in the culture supernatant only when the cells were co-cultured with *P. acnes*. Either anti-CAMP factor serum or desipramine, a selective ASMase inhibitor, significantly abrogated the *P. acnes*-induced cell death of HaCaT and RAW264.7 cells. Intradermal injection of ICR mouse ears with live *P. acnes* induced considerable ear inflammation, macrophage infiltration, and an increase in cellular soluble ASMase. Suppression of ASMase by systemic treatment with desipramine significantly reduced inflammatory reaction induced by intradermal injection with *P. acnes*, suggesting the contribution of host ASMase in *P. acnes*-induced inflammatory reaction *in vivo*. Vaccination of mice with CAMP factor elicited a protective immunity against *P. acnes*-induced ear inflammation, indicating the involvement of CAMP factor in *P. acnes*-induced inflammation. Most notably, suppression of both bacterial CAMP factor and host ASMase using vaccination and specific antibody injection, respectively, cooperatively alleviated *P. acnes*-induced inflammation.

**Conclusions/Significance:**

These findings envision a novel infectious mechanism by which *P. acnes* CAMP factor may hijack host ASMase to amplify bacterial virulence to degrade and invade host cells. This work has identified both CAMP factor and ASMase as potential molecular targets for the development of drugs and vaccines against acne vulgaris.

## Introduction

*Propionibacterium acnes* (*P. acnes*), a Gram-positive, anaerobic and lipophilic bacterium, plays an important role in inflammatory acne vulgaris. This disease is the most common disorder of the human skin, afflicting up to 80% of individuals at some point in their lives. Over-proliferation of *P. acnes* can be found in the microcomedone, which is the precursor of acne vulgaris characterized by hyperkeratinization, formation of a keratin plug, and increase in sebum secretion by the sebaceous gland [1]–[4]. The initial event in the inflammation of severe acne is the disruption of follicular epithelium by this overgrowth of *P. acnes*, allowing the bacteria in the microcomedone to spread in contact with various skin and immune cells such as keratinocytes and macrophages, thereby triggering granulomatous reactions of inflammatory acne [5]–[7]. *P. acnes* stimulates the production of pro-inflammatory cytokines, including interleukins -1β, -8, -12, and tumor necrosis factor-α, via toll-like receptor 2 [8]–[10].

Hemolysis has been employed by numerous bacterial pathogens to degrade, invade host cells, and to resist the host immune attack. This is achieved through various mechanisms such as enzymatic and/or pore formation activities targeting the host cell membranes [11]. When *P. acnes* is grown on a sheep blood agar plate in close proximity to β-hemolytic microorganisms, such as *Staphylococcus aureus* (*S. aureus*) and *Clostridium perfringens* [12], it synergistically enhances hemolysis similar to the classical Christie, Atkins, Munch-Peterson (CAMP) reactions first described by Christie and co-authors [13]. The CAMP reactions occur in various bacterial interactions. For example, in *Streptococcus agalactiae* (also named as group B streptococci, GBS) and *Bartonella henselae* (*B. henselae*), CAMP reactions are induced by the combination of CAMP factor co-hemolysin, which is a secreted pore-forming toxin, and sphingomyelinase (SMase) derived from other bacterial partners such as *S. aureus* [14], [15]. GBS CAMP factor itself has weak hemolytic activity on the erythrocytes, but pre-treating the cells with SMase enhances it's activity [14]. *S. aureus* SMase hydrolyzes sphingomyelin on the erythrocyte membranes to ceramide, which renders the cells more susceptible to the hemolytic activity of GBS CAMP factor [14]. The entire genomic sequence of *P. acnes* includes numerous genes whose products are involved in degrading host molecules [16]–[18]. Particularly, *P. acnes* carries five genes encoding CAMP factor homologs [19]. One of the CAMP factor homologs (CAMP factor 2, accession number: *gi*/50842175) shows high identity (33%) in amino acid sequence to the GBS CAMP factor.

Due to its co-hemolytic activity on erythrocytes *in vitro*, CAMP factor has been proposed as a virulence factor for various pathogens including GBS, *Streptococcus uberis* and *B. henselae* [14], [15], [20], [21]. However, the significance of erythrocyte lysis by the CAMP reaction *in vivo* remains unclear and the cytotoxic effects of CAMP factor on other cell types are not examined. In addition to its co-hemolytic activity, GBS CAMP factor was reported to bind to the Fc region of immunoglobulins G (IgG) and M in a manner similar to Protein A of *S. aureus*, resulting in the alternative nomenclature, Protein B [22]. However, some recent evidence contradicted this observation [23]. Hensler and co-workers demonstrated that full virulence was retained in a CAMP factor knockout GBS mutant strain [24]. Therefore, the involvement of CAMP factor co-hemolysin in the virulence of pathogens remains controversial and not clear.

So far no bacterial source of SMases has been found in acne lesions. *Staphylococcus epidermidis* is one of the major bacteria isolated from acne lesions besides *P. acnes*, but it does not generate a CAMP reaction with *P. acnes* (data not shown). On the other hand, *S. aureus* expressing SMase can be found on the skin and is frequently involved in cutaneous infections [25], but is rarely found in acne lesions [26]. These facts suggest that the CAMP reaction between *P. acnes* CAMP factor and other bacterial SMases may be insignificant for the virulence of *P. acnes*. However, SMases have been widely isolated and characterized from bacteria, yeast and various tissues as well as biological fluids of mammals [27]. In spite of low identity between bacterial and mammalian SMases, the amino acid sequences share a number of conserved residues, suggesting a common catalytic mechanism [28], [29]. Here we hypothesize that *P. acnes* benefits from a host SMase that amplifies the CAMP factor-mediated virulence of *P. acnes*. To test this hypothesis, we studied the involvement of a host SMase in CAMP factor-mediated virulence of *P. acnes* both *in vitro* and *in vivo*.

## Results

### Recombinant CAMP Factor of *P. acnes* Is Biologically Active and Displays a CAMP Reaction

**Figure 1:**
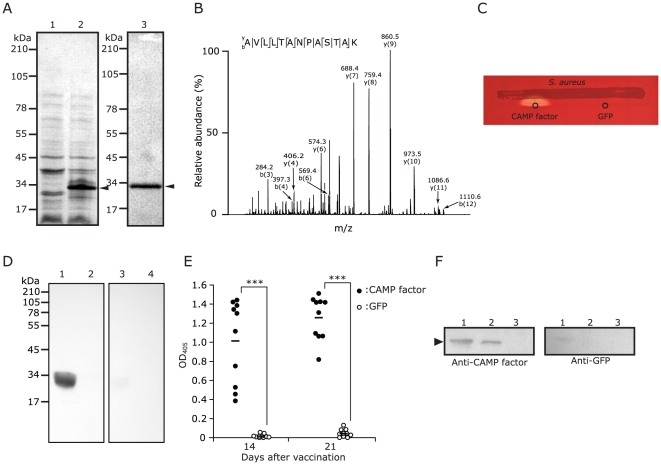
Characterization of *P. acnes* CAMP factor. (A) Recombinant CAMP factor (arrowheads) of *P. acnes* was expressed in *E. coli*. The protein expression was incubated without (lane 1) or with (lane 2) IPTG. Purified recombinant CAMP factor is shown (lane 3). (B) The identity of recombinant CAMP factor was analyzed by NanoLC-LTQ MS/MS mass spectrometry. A sequenced internal peptide (AVLLTANPASTAK) of CAMP factor is presented. (C) Co-hemolytic activity of recombinant CAMP factor was examined on a sheep blood agar plate. Recombinant CAMP factor (2.5 µg) or a GFP control protein (2.5 µg) was spotted beside the *S. aureus* streak. (D) Immunogenicity of CAMP factor was evaluated by Western blotting. ICR mice were intranasally vaccinated with UV-inactivated *E. coli* over-expressing CAMP factor or GFP. Sera were collected 14 days after the vaccination. Anti-CAMP factor (1∶2,000 dilution; lanes 1 and 2) or anti-GFP antiserum (lanes 3 and 4) was reacted with recombinant CAMP factor (0.2 µg; lanes 1 and 3) or GFP (lanes 2 and 4). (E) The antibody titer of CAMP factor was quantified by ELISA. The antisera (1∶10,000 dilution) were reacted with purified recombinant CAMP factor immobilized on a microtiter ELISA plate. The captured antibodies were detected by an OptEIA™ Reagent Set consisting of a goat-anti-mouse IgG (H+L)-HRP conjugate. The OD of each well was measured at 450 nm. Horizontal bar represents average of 10 individual assays. (F) CAMP factor was detectable in *P. acnes* culture medium by Western blotting. Recombinant CAMP factor (0.2 µg; lane 1) as a positive control, *P. acnes* culture medium (70 µg; lane 2), and RCM (70 µg; lane 3) as a negative control were reacted with mouse anti-CAMP factor antiserum (1∶1,000 dilution, left panel) or anti-GFP antiserum (right panel). The 6×HN tag linked to recombinant CAMP factor was removed by enterokinase before loading into a SDS-PAGE.

To express the CAMP factor, *E. coli* competent cells transformed with an expression plasmid containing an insert encoding *P. acnes* CAMP factor were incubated with Isopropyl-β-D-thiogalactoside (IPTG). A protein band with 32.4 kDa corresponding to the molecular mass of CAMP factor plus a 6×NH fusion protein was detected in the insoluble fraction of IPTG-induced *E. coli* (Figure 1A, left panel). A purified CAMP factor was obtained by using a TALON resin column (Figure 1A, lane 3) and sequenced by a NanoLC-LTQ MS/MS mass spectrometer after in-gel trypsin digestion (Figure 1B). Nine peptides were fully sequenced and matched well with internal amino acids of *P. acnes* CAMP factor (accession number: *gi*/50842175) (data not shown). A sequenced peptide (AVLLTANPASTAK; 147–158 amino acid residues) of CAMP factor is presented (Figure 1B), validating the expression and purification of recombinant CAMP factor. A conventional CAMP reaction was utilized to examine the biological activity of the recombinant CAMP factor. As shown in Figure 1C, a co-hemolysis was observed when recombinant CAMP factor was spotted adjacent to the SMase-expressing *S. aureus* on a sheep blood agar plate. Because *P. acnes* carries five genes encoding CAMP factor homologs [19], we examined co-hemolysis activity of another CAMP factor homolog (CAMP factor 4, accession number: *gi*/50840313), which shares high amino acid sequence identity (29%)to the GBS CAMP factor, however no activity was observed (Figure S1, Text S1). Other CAMP factor homologs (1, 3 and 5) show lower identity in amino acid sequences to the GBS CAMP factor. Thus, CAMP factor homolog 2 was used for this study.

### CAMP Factor Is Immunogenic When Mice Are Immunized with *E. coli* Over-Expressing CAMP Factor

To examine the immunogenicity of CAMP factor, we immunized ICR mice intranasally with UV-inactivated *E. coli* over-expressing CAMP factor or green fluorescence protein (GFP) (a control protein). Antibody (IgG) to CAMP factor was detected 14 days after immunization by a Western blot analysis (Figure 1D). The immunoreactivity to CAMP factor was undetectable in the GFP-immunized mice. ELISA analysis showed a significant increase in antibody titers 14 and 21 days after immunization (Figure 1E). Twenty-one days after immunization, the titer of anti-CAMP factor IgG in the serum from the CAMP factor-immunized mice was greater than 100,000 while the titer from the GFP-immunized mice was less than 100.

### *P. acnes* CAMP Factor Is a Secretory Protein

The supernatant of *P. acnes* cultures from logarithmic growth phase was concentrated and subjected to Western blotting with antiserum obtained from mice immunized with UV-inactivated *E. coli* over-expressing CAMP factor or GFP. In the immunoreaction to anti-CAMP factor antiserum, we detected a single band (Figure 1F, left panel, lane 2) at the position corresponding to recombinant CAMP factor that had been treated with enterokinase to remove 6×NH tag (Figure 1F, left panel, lane 1). The band was undetectable in the concentrated Reinforced Clostridium Medium (RCM) that was used for *P. acnes* culture (Figure 1F, left panel, lane 3). As a contrast, no bands were detected when the supernatant of *P. acnes* cultures was immunoreacted to anti-GFP antiserum (Figure 1F, right panel).

### CAMP Factor Is Cytotoxic to Keratinocytes and Macrophages

**Figure 2:**
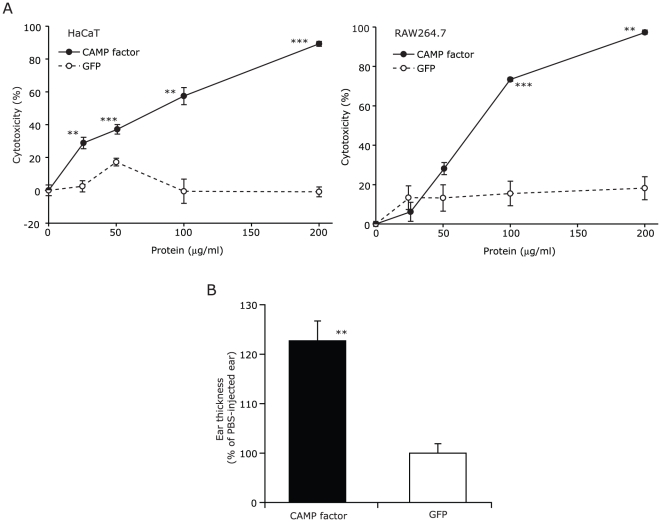
Virulence of *P. acnes* CAMP factor. (A) Cytotoxicity of recombinant CAMP factor was examined using HaCaT and RAW264.7 cells. The cells were incubated with recombinant CAMP factor or GFP at the indicated concentrations at 37°C for 18 hr. After the incubation, cytotoxicity was measured as described in Experimental Procedures. The data represent mean ± standard error (SE) (*n* = 6, *p*<0.005** and *p*<0.0005*** by Student's *t*-test, CAMP factor vs. GFP control). (B) Intradermal injection with CAMP factor induced the ear thickness increase of ICR mice. The left ear was intradermally injected with 20 µl of recombinant CAMP factor (10 µg) or GFP (10 µg) in PBS. Right ear received an equal volume of PBS. The ear thickness was measured using a micro caliper 24 hr after the injection and normalized to that of the PBS-injected ears. The data represented as mean ± SE (*n* = 4, *p*<0.005** by Student's *t*-test).

In the event of granulomatous type of severe acne inflammation, *P. acnes* escapes from the ruptured follicular wall and interacts with various skin cells such as keratinocytes and phagocytic cells such as macrophages. To explore the cytotoxicity of CAMP factor, a human HaCaT keratinocyte cell line and a murine RAW264.7 macrophage cell line were treated with various concentrations of recombinant CAMP factor or GFP. It was found that the treatment with CAMP factor resulted in dose-dependent cytotoxicity in both HaCaT and RAW264.7 cells (Figure 2A). To examine the virulence of *P. acnes* CAMP factor *in vivo*, mouse ear was injected intradermally with recombinant CAMP factor or GFP. Injection of CAMP factor, but not GFP, for 24 hr induced a significant increase in ear thickness (Figure 2B), demonstrating the virulence of *P. acnes* CAMP factor in an inflammatory reaction.

### Both CAMP Factor and Host ASMase Are Involved in *P. acnes* Virulence

**Figure 3:**
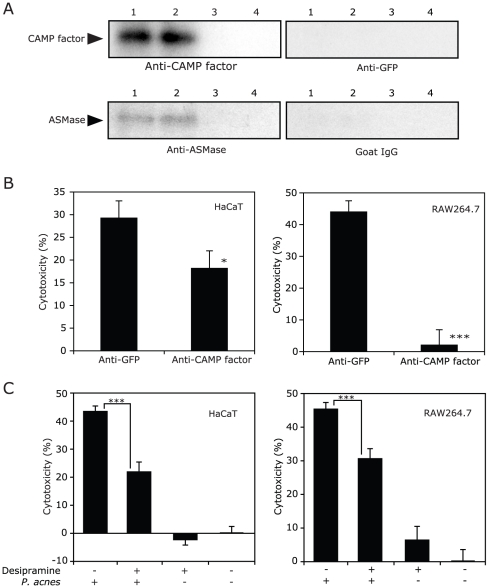
Synergy of *P. acnes'* CAMP factor with a cellular ASMase in promoting cytotoxicity. (A) CAMP factor and ASMase were detectable by Western blotting in the medium of co-culturing *P. acnes* and the cells. The HaCaT (lanes 1 and 3) or RAW264.7 (lanes 2 and 4) cells were cultured together with *P. acnes* (MOI = 1∶10) (lanes 1 and 2) or without *P. acnes* (lanes 3 and 4) in a serum-free medium at 37°C for 14 hr. CAMP factor and ASMase secreted into the culture media were detected by mouse anti-CAMP factor antiserum and goat anti-ASMase IgG, respectively. (B) *P. acnes*-mediated cytotoxicity was neutralized by anti-CMAP factor antiserum *in vitro*. HaCaT or RAW264.7 cells were co-cultured with *P. acnes* (MOI = 1∶10) for 14 hr in the presence of mouse anti-CAMP factor or anti-GFP antiserum (2.5% v/v). (C) ASMase inhibitor suppressed *P. acnes*-mediated cytotoxicity *in vitro*. HaCaT or RAW264.7 cells were cultured without or with *P. acnes* (MOI = 1∶10) in medium containing desipramine (10 µM), a selective ASMase inhibitor, or the equal volume of PBS at 37°C for 14 hr. After incubation, cytotoxicity was calculated as described in Experimental Procedures. The data represent as mean ± SE (*n* = 10, *p*<0.05* and *p*<0.0005*** by Student's *t*-test).

To examine whether acid SMase (ASMase) is released from host cells in the presence of *P. acnes*, HaCaT and RAW264.7 cells were cultured with or without *P. acnes* for 14 hr, respectively. After incubation, the culture supernatant was subjected to Western blotting, probing with an anti-CAMP factor antiserum and an anti-ASMase IgG. The CAMP factor and ASMase were simultaneously present in the culture supernatant only when the cells were co-cultured with *P. acnes* (Figure 3A, lanes 1 and 2). The human (gi/179095) and mouse (gi/21961231) mature ASMases with similar molecular weights share greater than 90% amino acid identity [30]. Neither of these ASMases was detected in the culture supernatant in the absence of *P. acnes* (Figure 3A, lanes 3 and 4). Furthermore, the ASMase was not detectable in the supernatant of *P. acnes* culture alone (data not shown). These findings indicate that both CAMP factor and ASMase are released when co-culturing *P. acnes* with host cells.

To examine the effect of neutralization of CAMP factor on *P. acnes*-induced cytotoxicity, HaCaT and RAW264.7 cells were co-cultured with *P. acnes* in the presence of anti-CAMP factor or anti-GFP antiserum. As shown in Figure 3B, in the presence of anti-GFP antiserum, *P. acnes* induced 29.3±3.8% and 44.0±3.4% cell death on HaCaT and RAW264.7 cells, respectively. The co-culture of anti-CAMP factor antiserum reduced *P. acnes*-induced cell death of HaCaT and RAW264.7 cells to 18.2±3.9% and 2.1±4.6%, respectively. To investigate the involvement of host ASMase in the cytotoxicity of *P. acnes*, cells were co-cultured with *P. acnes* in the presence of desipramine, a selective ASMase inhibitor, or an equal volume of PBS as a control (Figure 3C). *P. acnes* alone induced 43.4±1.4% and 45.4±1.8% cell death on HaCaT and RAW264.7 cells, respectively. The addition of desipramine significantly reduced *P. acnes*-induced cell death on both cells to 21.9±3.2% and 30.6±2.7%, respectively. These results clearly show that both bacterial CAMP factor and cell ASMase are part of the cause of cytotoxicity induced by *P. acnes*.

**Figure 4:**
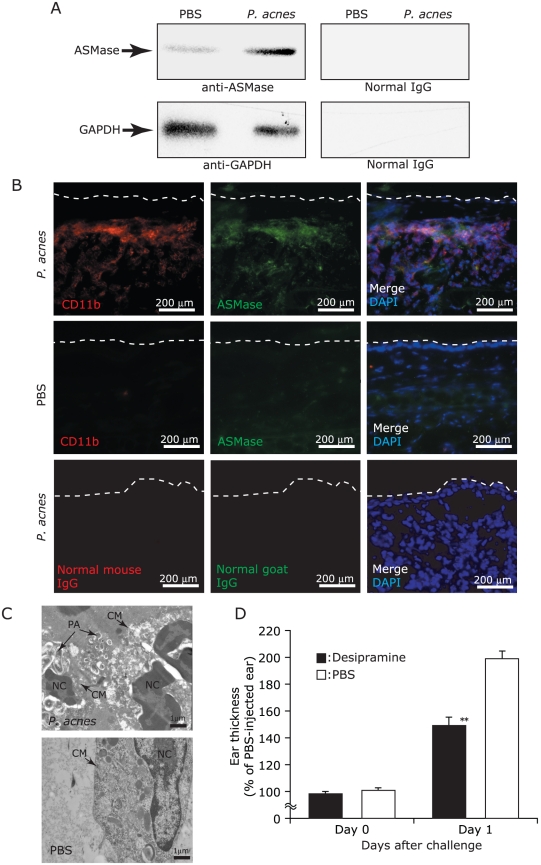
Involvement of host ASMase in the virulence of *P. acnes in vivo*. (A) The level of soluble ASMase in mouse ear was elevated 24 hr after bacterial injection. Ears of ICR mice were injected intradermally with *P. acnes* (1×10^7^ CFU; left ear) or PBS (right ear) for 24 hr. Ear tissues were homogenized in PBS and centrifuged. The supernatant (1 µg) was subjected to Western blotting using anti-ASMase IgG and anti-GAPDH IgG. Normal goat and mouse IgG were used as negative controls. (B) *P. acnes* injected into mouse ear recruited CD11b^+^ macrophages that highly expressed ASMase. Frozen sections of mouse ears obtained 24 hr after bacterial injection were stained with biotinylated anti-mouse CD11b IgG, a conventional macrophage marker, and TRITC-streptavidin conjugate (red), followed by goat anti-ASMase IgG and anti-goat IgG-TRITC conjugate (green). Biotinylated normal mouse IgG and normal goat IgG were used as isotype control antibodies. The nuclei were stained with DAPI (blue). Broken lines represent the outlines of ear sections. Bar = 200 µm. (C) Transmission electron microscopy (10,000× magnification) was utilized to visualize colonized *P. acnes* and ruptured cell membranes in mouse ears injected with *P. acnes* or PBS. PA, *P. acnes*; CM, cell membrane; NC, nucleus. Bar = 1 µm. (D) Systemic pre-treatment of ICR mice with a selective ASMase inhibitor alleviated *P. acnes*-induced increase in ear thickness. ICR mice were injected intraperitoneally with desipramine (20 mg/kg mouse) (solid bars) or an equal volume of PBS (open bars) 30 min prior to bacterial injection. Live *P. acnes* (1×10^7^ CFU) and an equal volume of PBS were injected intradermally into left ear and right ear of the mice, respectively. The ear thickness was measured before and 24 hr after bacterial injection, and was normalized to that of the PBS-injected controls. The data represent as mean ± SE (*n* = 3, *p*<0.005** by Student's *t*-test).

To assess the contribution of host ASMase in *P. acnes* cytotoxicity *in vivo*, the ears of ICR mice were injected intradermally with *P. acnes* or PBS for 24 hr and subsequently excised for the following studies. First, the supernatant of ear homogenate was immunoreacted to anti-ASMase IgG in a Western blot analysis (Figure 4A). A single band corresponding to ASMase with a molecular weight of about 60 kDa was detected. *P. acnes* injection significantly increased the amount of soluble ASMase in the ear in comparison with PBS injection. Second, frozen sections of mouse ears were stained with anti-mouse CD11b IgG (red), a macrophage positive marker, followed by anti-ASMase IgG (green) (Figure 4B). The merged images (pink) evidently illustrated that ASMase was predominantly expressed in the infiltrating CD11b^+^ macrophages, suggesting that injection of *P. acnes*, but not PBS, recruited the infiltration of macrophages. Third, transmission electron microscopy showed that *P. acnes* was phagocytosed in macrophage-like cells as well as distributed in the extracellular space 24 hr after bacterial injection (Figure 4C). Furthermore, ruptured cell membranes were observed exclusively in the *P. acnes*-, but not PBS-injected ears, indicating that intradermal *P. acnes* injection induces the infiltration of ASMase-expressed macrophages. Lastly, measurement of ear thickness showed that *P. acnes*-induced ear swelling was significantly alleviated when mice were pretreated systemically with desipramine for 30 min (Figure 4D), supporting that host ASMase engages *P. acnes*-induced ear inflammation. All these findings concurrently demonstrate the co-contribution of CAMP factor and ASMase to *P. acnes* virulence in terms of cytotoxicity and inflammation.

### Inhibition of Both Bacterial CAMP Factor and Host ASMase Cooperatively Suppress *P. acnes*-Induced Inflammation

**Figure 5:**
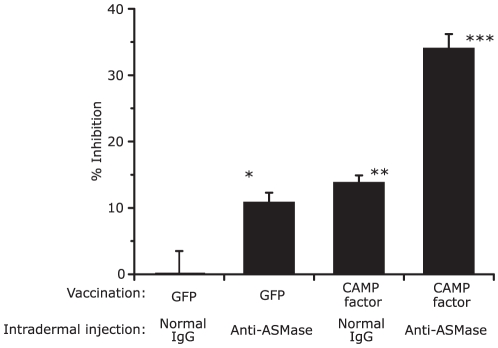
Combination of CAMP factor vaccination and intradermal injection with anti-ASMase IgG cooperatively suppressed *P. acnes*-induced ear inflammation. ICR mice were vaccinated with UV-inactivated *E. coli* over-expressing CAMP factor (solid and grey bars) or GFP (hatched and open bars) in a 3-week interval. Two weeks after the second boost, *P. acnes* was injected intradermally into the ears of vaccinated mice in the same manner as described in Figure 4. Thirty min after bacterial injection, ears at the same site received *P. acnes* (left ear) or PBS (right ear) were subsequently injected with goat anti-ASMase IgG (4 µg) (*n* = 8). As a control, an equal volume of normal goat IgG in PBS was injected intradermally into both ears (*n* = 8). The change in ear thickness was measured 24 hr after the bacterial injection and normalized to that of the PBS-injected ears. The data was expressed as percent inhibition of *P. acnes*-induced increase in ear thickness determined by comparison with that in the mice treated with GFP vaccination combined with normal IgG injection. The data represent as mean ± SE (*p*<0.05*, *p*<0.005**, *p*<0.0005*** by Student's *t*-test).

ICR mice were vaccinated intranasally with UV-inactivated *E. coli* over-expressing CAMP factor or GFP. *P. acnes* or PBS was injected intradermally into the ears of vaccinated mice for 24 hr. Thirty minutes after bacterial injection, the left ear, which received *P. acnes*, was subsequently injected with anti-ASMase IgG or normal goat IgG, while the right ear, which received PBS, was continuously injected with an equal volume of PBS. In comparison with increased ear thickness in the mice treated with GFP vaccination combined with normal IgG injection, *P. acnes*-induced ear swelling was reduced for the combination of GFP vaccination with anti-ASMase IgG injection (20.7±2.9% inhibition) and the combination of CAMP factor vaccination with normal goat IgG (25.8±1.9% inhibition). More importantly, the combination of CAMP factor vaccination with anti-ASMase IgG injection reduced *P. acnes*-induced ear swelling (60.3±3.9% inhibition) (Figure 5). This result demonstrates that a synergistic abrogation of *P. acnes*-induced inflammation may occur when both *P. acnes* CAMP factor and host ASMase are suppressed. The result also suggests a cross-talk between *P. acnes* CAMP factor and host ASMase may exist that enhances bacterial virulence.

## Discussion

The hemolysis mainly caused by hemolysins is thought to be a virulence activity of numerous microbial pathogens to degrade tissues, invade host cells, disseminate themselves, and escape from the host immune attack. Microbial hemolysins generally possess the capability to lyse erythrocytes *in vitro*, but many of them are toxic to other cell types as well [11]. We observed that *P. acnes* secretes CAMP factor as an exotoxin (Figure 1F). Although the hemolytic action of bacterial CAMP factor has been demonstrated on erythrocytes and artificial plasma membranes [14], [15], [31], little attention has been paid to the cytotoxicity of CAMP factor on other cell types. Here, we examined cytotoxic activity of *P. acnes* CAMP factor on host cells, and its physiologic relevance to the pathogenicity of *P. acnes*, which is highly relevant to severe inflammatory acne vulgaris. In the case of severe acne vulgaris, the keratinocytes and macrophages are two major target cells when *P. acnes* escapes from ruptured follicular wall. Our data demonstrated that *P. acnes* CAMP factor is toxic to both human HaCaT keratinocytes and murine RAW264.7 macrophages (Figure 2A). However, we could not conclude that CAMP factor itself exerts cytotoxic effects on these cells as they have ASMase (Figure 3). Synergistic effect of recombinant CAMP factor and cellular ASMase might be involved in the dose-dependent cytotoxic effect. There have been only a few studies showing that CAMP factor is a potential virulence factor of pathogen *in vivo*. A high dose of partially purified CAMP factor from GBS was lethal to rabbits and mice when it was injected intravenously [32]. Mice that had been infected with sublethal doses of GBS developed fatal septicemia after receiving repeated injections with purified CAMP factor [22]. We demonstrated that intradermal injection of the mouse ears with recombinant CAMP factor of *P. acnes* induced ear swelling (Figure 2B), indicating that CAMP factor is involved in *P. acnes*-induced inflammation *in vivo*. Furthermore, we demonstrated that intradermal injection of mouse ears with live *P. acnes* induces tremendous infiltration of ASMase-expressed CD11b^+^ macrophages (Figure 4B and C), consistent with our previous observation that injection of live *P. acnes* into an implanted tissue chamber-imitated acne microenvironment recruited remarkable CD11b^+^ macrophages [33].

Several different forms of mammalian SMases have been identified, including soluble endosomal/lysosomal SMase present in all mammalian tissues, plasma membrane-associated neutral SMase predominantly present in the central nervous system, and cytosolic SMase [34]–[37]. These enzymes catalyze the hydrolytic cleavage of sphingomyelin on the cell membrane to ceramide in the same catalytic mechanism as bacterial SMases. The released ceramide, in turn, can act as a cellular signal to trigger various activities such as apoptosis, differentiation, and proliferation [38]. The activity of the SMases are regulated by a wide range of extracellular signaling; growth factors, cytokines, neurotransmitters, hormones, and stresses, such as ultraviolet and reactive oxygen species [35]. Ubiquitously-expressed ASMase exerts important functions during the innate immune response to infectious pathogens [39]. Therefore, we focused on the interaction of host ASMase and bacterial CAMP factor as related to virulence of *P. acnes*. ASMase was released from the host cells when exposing to *P. acnes* (Figure 3A). The cytotoxicity of *P. acnes* was dramatically neutralized in the presence of mouse anti-CAMP factor antiserum or a specific ASMase inhibitor, desipramine [40], (Figure 3B and C), suggesting that *P. acnes* CAMP factor and cell ASMase co-contribute to the virulence of *P. acnes*. However, anti-CAMP factor antiserum only partially neutralized the cytotoxicity of *P. acnes* on HaCaT cells, suggesting that there are other cytotoxic mechanisms in addition to the CAMP factor for keratinocytes. Because desipramine displays an anti-depressant property as well as anti-ASMase inhibition [41], [42], we examined the specificity of desipramine on CAMP factor-mediated cytotoxicity. Desipramine significantly suppressed the cytotoxicity of *P. acnes* CAMP factor, but did not influence the cytotoxicity associated with *S. aureus* α-toxin, used as a control toxin (Figure S2, Text S1). The data also indicates that host ASMase is specifically involved in virulence of *P. acnes* CAMP factor, but not in those of other toxins. In addition, desipramine may have a therapeutic potential for acne by blocking the synergistic cytotoxicity of *P. acnes* CAMP factor and host ASMase.

Inhibition of both *P. acnes* CAMP factor and host ASMase synergistically suppressed the *P. acnes*-induced inflammation (Figure 5), indicating a cross-talk between CAMP factor and ASMase. These data suggest an opportunity to block the cross-talk as an anti-inflammatory treatment of acne. To our knowledge, this may be the first study to examine the therapeutic potential of blocking CAMP factor as a treatment of infection. In future experiments, however, it will be necessary to investigate whether host ASMase enhances bacterial CAMP factor toxicity *in vitro* and to use ASMase deficient mice and a CAMP factor-deficient mutant of *P. acnes* to completely confirm the existence of cross-talk.

Lang and co-authors have reported that GBS CAMP factor binds to glycosylphosphatidylinositol (GPI)-anchored proteins on the cell membrane of erythrocytes, which act as cell surface receptors for this toxin [31], [43]. The interaction between the GBS CAMP factor and the GPI-anchored proteins further induce the formation of oligomeric pores in sheep erythrocyte membranes [14]. The amount of GPI-anchored proteins is augmented by the reduction of sphingolipid levels on the cell membrane [44]. Since GPI-anchored proteins are found ubiquitously in mammalian cells [45], the same mechanism may be involved in the cytotoxic reaction of *P. acnes* CAMP factor to keratinocytes and macrophages. Indeed, removal of sphingomyelin on the cell membranes by pre-treating the cells with bacterial SMase increased the cell susceptibility to *P. acnes* CAMP factor (Figure S3, Text S1).

The contribution of host ASMase to *P. acnes* virulence was also examined. Ears of ICR mice were injected intradermally with live *P. acnes* [46], [47]. The amount of soluble ASMase increased in the ears after injection with *P. acnes* (Figure 4A). The model of granulomatous reactions in mouse ears (Figure 4B and C) recapitulates severe inflammatory acne in humans, in which numerous *P. acnes* has been observed inside phagosomes of an infiltrating macrophage [6]. It has been reported that *P. acnes* resists killing by phagocytes and is able to survive in macrophages [48]. GBS β-hemolysin/cytolysin, a pore-forming exotoxin, has been demonstrated to contribute to the subversion of phagocytic host immune defenses [49]. During the intracellular life cycle of *Listeria monocytogenes*, a pore-forming toxin named listeriolysin O is largely responsible for mediating phagosomal membrane rupture to allow the pathogen escaping from the phagosome into the host cytosol [50]. Lysosomal ASMase plays an important role in macrophage killing bacteria at the early stage of phagocytosis [51], [52] and enables proper fusion of late phagosomes with lysosomes, which is crucial for efficient transfer of lysosomal antibacterial hydrolases into phagosomes [53]. The results in this paper highlights the possibility that phagocytosed *P. acnes* in the macrophage may take advantage of the host lysosomal ASMase to enhance the toxicity of CAMP factor and thus escape from phagosomes. This may also explain the possible mechanism of *P. acnes* resistance against phagocytosis. Indeed, we observed a number of macrophages in the *P. acnes*-injected ear, many of which had cell membranes ruptured by colonizing *P. acnes* (Figure 4C). In agreement with our observation in Figure 3A, infection by Salmonella or *E. coli* triggered an early surge of the extracellular secretion of ASMase from macrophages [51]. *P. acnes* may shrewdly utilize the secreted ASMase from macrophages to escape from the host cells and spread invasively from cell to cell. Our results also envision that *P. acnes* CAMP factor hijacks host ASMase to amplify its virulence.

We have recently developed acne vaccines using UV-killed *P. acnes* [54] or a cell-wall anchored sialidase [55] as an antigen. These vaccines will benefit patients with severe acne or other *P. acnes*-associated diseases such as polymer (device)-associated diseases, sepsis, toxic shock syndrome, endocarditis, osteomyelitis and various surgery infections [56]. It has been reported that a single intraperitoneal injection of phenol-treated *P. acnes* into mice showed non-specific resistance against subsequent lethal doses of an intraperitoneal challenge of *S. aureus* and *Streptococcus pyogenes* [57]. This suggests that using killed *P. acnes* as a vaccine may lack bacterial specificity. Instead, vaccines that target the secreted virulence factors such as CAMP factor may work in a different way from surface-targeted vaccines, thereby eliciting the bactericidal antibodies to eradicate bacteria directly. Recently, it has been demonstrated that inhibition of microbial secreted virulence factors present less selective pressure for the generation of resistance [58]. *P. acnes* is a ubiquitous commensal on the human body and can become pathogenic in anaerobic acne lesions [59]. Since the *P. acnes* CAMP factor and host ASMase are secreted locally in acne microenvironment, the application of vaccines that target the secreted CAMP factor instead of other bacterial surface proteins and antibodies that neutralize ASMase may be able to locally suppress the *P. acnes* virulence while not killing the bacteria or impacting the bacterial commensalism in other locations of the body.

## Materials and Methods

### Ethics statement

All animal protocols were reviewed and approved by the University of California San Diego (approval number: S09330) and the Veterans Affairs San Diego Healthcare System subcommittee on animal studies (approval number: 08-308).

### Bacterial culture

*P. acnes* (ATCC 6919) was as described by our previous reports [54], [55], [60]. S. aureus 113 (ATCC 35556) was cultured on Tryptic soy broth (TSB) agar plates. Bacteria isolated from a single colony were inoculated in TSB at 37°C overnight. Bacterial pellets were harvested by centrifugation at 5,000 g for 10 min.

### Molecular cloning and expression of recombinant CAMP factor

A PCR product encoding a putative mature protein (29-267 amino acid residues) of CAMP factor (accession number: *gi*/50842175) was generated by using *P. acnes* genomic DNA as a template, the forward PCR primer (5′-TAAGGCCTCTGTCGACGTCGAGCCGACGACGACCATCTCG-3′) and the reverse PCR primer (5′-CAGAATTCGCAAGCTTGGCAGCCTTCTTGACATCGGGGGAG-3′). The amplified DNA products were inserted into the In-Fusion Ready pEcoli-Nterm 6×HN vector (Clontech Laboratories, Mountain View, CA) and transformed into competent cells [*Escherichia coli* (*E. coli*), BL21 (DE3), Invitrogen, Carlsbad, CA]. To express GFP, a pEcoli-Nterm-GFP plasmid (Clontech Laboratories Inc.) was transformed. IPTG (1 mM) was used to induce protein synthesis. The expressed protein was purified in a denaturing condition with a TALON Express Purification Kit (Clontech Laboratories Inc.). The purified protein was refolded as described previously [55].

### Protein identification via mass spectrometry (MS)

In-gel digestion with trypsin and protein identification via a Nano liquid chromatography linear trap quadrupole mass spectrometry (NanoLC-LTQ MS/MS) analysis were performed as described previously [61].

### Co-hemolytic activity of CAMP factor

Co-hemolytic reaction of recombinant CAMP factor was detected on a sheep blood agar plate according to the previous method [62] with minor modification. *S. aureus* 113 (ATCC 35556) [10 µl, 2×10^7^ colony forming unit (CFU)/ml], used as a source of SMase, was streaked on an agar plate. Recombinant CAMP factor or GFP (2.5 µg) was spotted beside the *S. aureus* streak grown at 37°C for 18 hr.

### Vaccination and titration of antibodies to CAMP factor

An 8-week-old female Institute Cancer Research (ICR) mouse strain (Harlan, Indianapolis, IN) was used in all animal experiments. ICR mice were housed according to institutional guidelines. The mice were intranasally vaccinated with UV-inactivated *E. coli* over-expressing CAMP factor or GFP. The use of a UV-irradiated *E. coli* vector eliminates the necessity of boosting [63].

To qualify the titer of antibody to CAMP factor, recombinant CAMP factor (5 µg/ml) was coated onto a 96-well ELISA plate (Corning, Lowell, MA). After blocking, antisera (1∶10,000 dilution) obtained from mice vaccinated with *E. coli* over-expressing CAMP factor or GFP were added to the wells and incubated for 2 hr. A goat anti-mouse IgG (H+L) IgG-horseradish peroxidase (HRP) conjugate (Promega, Madison, WI) (1∶5,000 dilution) was added and incubated for 2 hr. HRP activity was determined with an OptEIA™ Reagent Set (BD Biosciences, San Jose, CA). The OD of each well was measured at 450 nm.

### Cell culture, cytotoxicity determination and neutralization assay

A human keratinocyte cell line, HaCaT [64], and a murine macrophage cell line, RAW264.7 (ATCC, Manassas, VA), was cultured in DMEM and RPMI 1640 medium, respectively, supplemented with 10% heat-inactivated fetal bovine serum (FBS). For determination of the cytotoxicity of CAMP factor, cells (1×10^5^/well) were incubated in a 96-well plate with recombinant CAMP factor or GFP in a 1% FBS-medium for 18 hr. After incubation, cell viability was determined by an acid phosphatase (ACP) assay [65] as described previously [54], [55]. The cytotoxicity of recombinant proteins was calculated as the percentage of cell death caused by Triton X-100 (0.1%, v/v).

To detect the release of CAMP factor and acid SMase (ASMase), HaCaT or RAW264.7 cells (5×10^5^/well) were co-cultured with or without *P. acnes* [5×10^6^ CFU/well; multiplicity of infection (MOI) = 1∶10] in a serum-free medium in a 24-well plate at 37°C for 14 hr. After centrifugation, the supernatant was filtrated with a 0.22 micro pore-size filter and then concentrated 10-fold using a 10 kDa cut-off ultrafiltration membrane (Amicon Inc., Beverly, MA). The concentrated supernatant (10 µg) was subjected to a 10% SDS-PAGE for Western blot analysis using mouse anti-CAMP factor antiserum and goat anti-ASMase IgG (Santa Cruz Biotechnology Inc., Santa Cruz, CA).

For neutralization assay, cells (1×10^5^/well) were co-cultured with *P. acnes* (1×10^6^ CFU/well; MOI = 1∶10) for 14 hr in the presence of anti-CAMP factor or anti-GFP antiserum (2.5%, v/v) in which complements were deactivated by heating. To examine the involvement of host ASMase in *P. acnes* virulence, cells were co-cultured with *P. acnes* in the presence or absence of desipramine (10 µM) (Sigma, St. Louis, MO), a cell-permeable selective ASMase inhibitor [40], for 14 hr. After incubation, the cytotoxicity of *P. acnes* was determined as described above.

### Intradermal injection of mouse ears with recombinant CAMP factor

Ears of ICR mice were injected intradermally with 20 µl of recombinant CAMP factor (0.5 mg/ml) or GFP in PBS. The contralateral ear received an equal volume of PBS. The ear thickness was measured using a micro caliper (Mitutoyo, Kanagawa, Japan) 24 hr after injection. The increase in ear thickness of CAMP factor- or GFP-injected ear was normalized to that of the PBS-injected ears.

### Detection of ASMase in mouse ears

Ears of ICR mice were injected intradermally with live *P. acnes* (1×10^7^ CFU/20 µl in PBS). The contralateral ear received an equal volume of PBS. Twenty four hr after bacterial injection, the ear was excised, punched with an 8 mm biopsy and homogenized in PBS. After centrifugation, the supernatant (1 µg) was subjected to a Western blot analysis using goat anti-ASMase IgG (0.2 µg/ml) (Santa Cruz Biotechnology, Inc.) followed by monoclonal anti-glyceraldehyde 3-phosphate dehydrogenase (GAPDH) IgG (2 µg/ml) (Fitzerald Inc., Concord, MA). Normal goat IgG or mouse IgG was used as a negative control.

### Transmission electron microscopy and fluorescence immunohistochemistry

Mouse ears were injected intradermally with live *P. acnes* or PBS as described above. Twenty four hr after bacterial injection, ears were excised and fixed in Karnovsky's fixative followed by 1% OsO_4_ in 0.1 M Na Cacodylate buffer, pH 7.4. After polymerization with epoxy resin, thin sections were prepared. Sections were examined at an accelerating voltage of 60 kV using a Zeiss EM10C electron microscope (Oberkochen, Germany).

For fluorescence immunohistochemistry, the *P. acnes*-injected ears were excised 24 hr after bacterial injection. Frozen sections were fixed in 10% formamide. After blocking with PBS containing 5% BSA and anti-mouse cluster of differentiation (CD) 16/CD32 IgG (5 µg/ml) (BD Biosciences), sections were then incubated with biotinylated anti-mouse CD11b IgG (5 µg/ml) (BD Biosciences), a macrophage marker, followed by goat anti-ASMase IgG (5 µg/ml) for 30 min. Biotinylated normal mouse IgG and normal goat IgG were used as isotype control antibodies. After that, sections were incubated with tetramethylrhodamine isothiocyanate (TRITC)-streptavidin conjugate (5 µg/ml) (ZYMED, Carlsbad, CA) or fluorescein isothiocyanate (FITC)-labeled anti-goat IgG (5 µg/ml) (Santa Cruz Biotechnology Inc.) for 30 min at room temperature before counterstaining with by 4′-6-Diamidino-2-phenylindole (DAPI) (Sigma).

### Effect of desipramine on *P. acnes*-induced inflammation *in vivo*

ICR mice were injected intraperitoneally with desipramine (20 mg in PBS/kg mouse). Because 86% of desipramine binds to plasma proteins [66], the dosage was determined to achieve a higher plasma concentration (about 1 mM as the average total blood volume of a mouse is about 77–80 ml/kg) than the concentration used for *in vivo* assay (10 µM). Injection of an equal volume of PBS serves as a control. Thirty minutes after desipramine treatment, the ears were injected intradermally with either live *P. acnes* or PBS. Ear thickness was measured before and 24 hr after bacterial injection, and was normalized to that of PBS-injected ears.

### Cooperative effects of CAMP factor-based vaccine and anti-ASMase IgG on the suppression of *P. acnes*-induced inflammation

ICR mice were vaccinated with inactivated *E. coli* over-expressing CAMP factor or GFP in a 3-week interval. Two weeks after the second boost, live *P. acnes* (1×10^7^ CFU/20 µl in PBS) was injected intradermally into the left ears of vaccinated mice. The same volume of PBS was injected into right ears of the same mice. Thirty minutes after injection, both ears were subsequently injected with goat anti-ASMase IgG (4 µg/20 µl) or normal goat IgG as a negative control. The ear thickness in the left ears was normalized to that of the PBS-injected right ears.

## Supporting Information

Text S1 (0.03 MB DOC)

Figure S1 Co-hemolytic activity of recombinant CAMP factor 4. Co-hemolytic reaction of recombinant CAMP factor 4 was examined on a sheep blood agar plate as described in Materials and Methods. Recombinant CAMP factor 4 or GFP (2.5 µg) was spotted beside the *S. aureus* streak grown at 37°C for 18 hr.
(0.02 MB PDF)

Figure S2 Desipramine suppressed cytotoxicity of *P. acnes* CAMP factor but not *S. aureus* α-toxin. HaCaT cells were incubated with CAMP factor (100 µg/ml), GFP (100 µg/ml), or α-toxin (20 µg/ml) in 1% FBS-medium for 18 hr in the presence or absence of desipramine (10 µM). After the incubation, cytotoxicity was measured as described in Experimental Procedures. The data represent mean ± standard error (SE) (*n* = 8, *p*<0.05* and *p*<0.0005*** by Student's *t*-test, desipramine vs. vehicle control).
(0.15 MB JPG)

Figure S3 Co-cytotoxic properties of CAMP factor and bacterial SMase *in vitro*. The HaCaT or RAW264.7 cells were pre-treated with *S. aureus* SMase (350 mU/ml) or an equal volume of PBS (vehicle) for 15 min and then incubated with 25 µg/ml of recombinant CAMP factor or GFP at 37°C for 18 hr. After the incubation, cell viability expressed as % of cytotoxicity was determined. The data are presented as mean ± SE (*n* = 6, *p*<0.0005*** by Student's *t*-test).
(0.18 MB JPG)
