# Supplementary material for: Propionibacterium acnes CAMP Factor and Host Acid Sphingomyelinase Contribute to Bacterial Virulence: Potential Targets for Inflammatory Acne Treatment
Source: PLoS One. 2011 Apr 12;6(4):e14797. doi: 10.1371/journal.pone.0014797 (PMC3075254; doi:10.1371/journal.pone.0014797)
Supplement: Text S1 — (0.03 MB DOC) [file pone.0014797.s001.doc]

**Text S1**

**EXPERIMENTAL PROCEDURES**

**Co-cytotoxic activity of CAMP factor and bacterial SMase.** The human keratinocyte cell line, HaCaT, and murine macrophage cell line, RAW264.7, were cultured in DMEM and RPMI1640 medium, respectively, supplemented with 10% heat-inactivated FBS, at 37^o^C under 5% (v/v) CO_2_. For determination of co-cytotoxic activity of SMase and CAMP factor, cells (1×10^5^/well) in a 96-well plate were pre-treated with *S. aureus* SMase (350 mU/ml, Sigma) or an equal volume of PBS (a vehicle control) in serum-free medium containing 10 mM MgCl_2_ for 15 min. After the pre-treatment, cells were washed with PBS and then incubated with CAMP factor (25 µg/ml) or GFP as a control in 1% serum-medium for 18 hr. Triton X-100 [0.1% (v/v)] was added to lyse all cells as 100% cytotoxicity. Cell viability was determined and cytotoxicity was calculated as described in the section of Experimental Procedures.

**Effect of desipramine on cytotoxicity of *P. acnes* CAMP factor and *S. aureus* α-toxin.** Desipramine, a selective ASMase inhibitor, and α-toxin from *S. aureus* (Toxin Technology Inc., Sarasota, FL) prepared in PBS. HaCaT cells were incubated with CAMP factor (100 µg/ml), GFP (100 µg/ml), or α-toxin (20 µg/ml) in 1% FBS-medium for 18 hr in the presence or absence of desipramine (10 µM). An equal amount of PBS was used as a negative control for desipramine. After the incubation, cell viability was determined and the cytotoxicity was calculated as the percentage of cell death caused by Triton X-100 (0.1%, v/v).
