# Supplementary figures and images for: Propionibacterium acnes CAMP Factor and Host Acid Sphingomyelinase Contribute to Bacterial Virulence: Potential Targets for Inflammatory Acne Treatment
Source: PLoS One. 2011 Apr 12;6(4):e14797. doi: 10.1371/journal.pone.0014797 (PMC3075254; doi:10.1371/journal.pone.0014797)

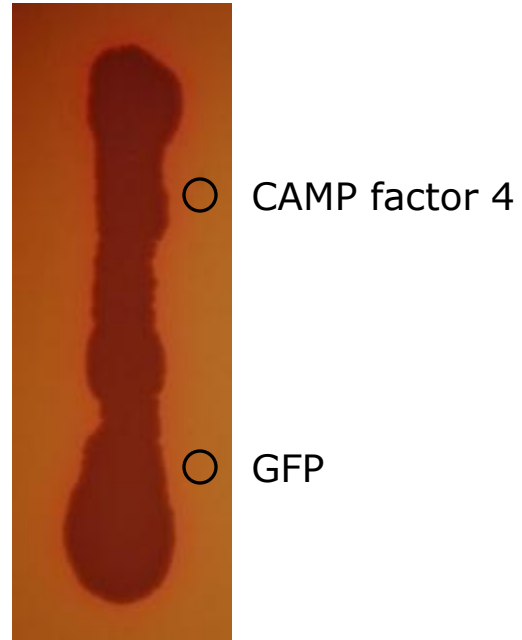

Figure S1

Supplement: Figure S1 — Co-hemolytic activity of recombinant CAMP factor 4. Co-hemolytic reaction of recombinant CAMP factor 4 was examined on a sheep blood agar plate as described in Materials and Methods. Recombinant CAMP factor 4 or GFP (2.5 µg) was spotted beside the S. aureus streak grown at 37°C for 18 hr. (0.02 MB PDF) [file pone.0014797.s002.pdf]

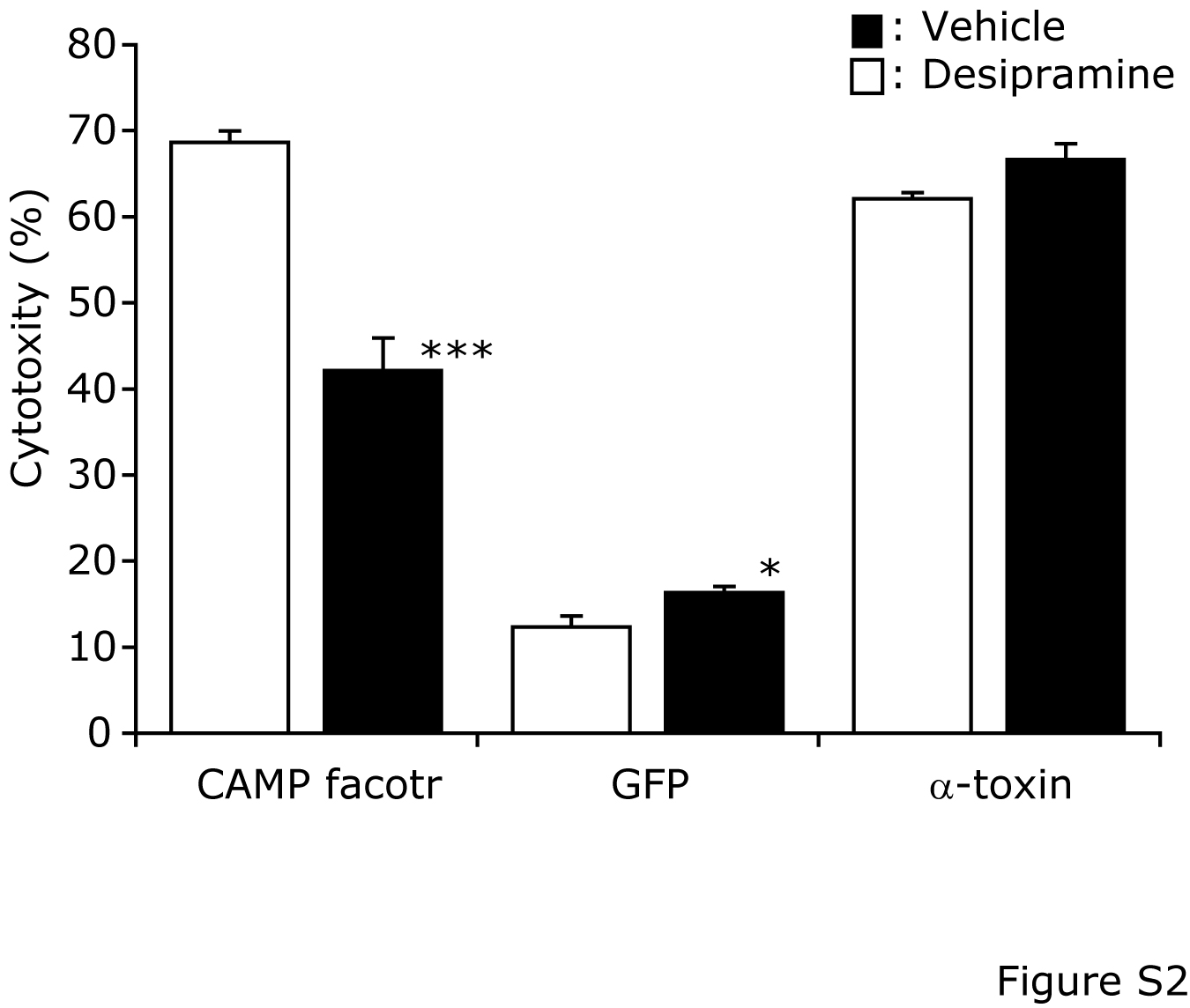

Supplement: Figure S2 — Desipramine suppressed cytotoxicity of P. acnes CAMP factor but not S. aureus α-toxin. HaCaT cells were incubated with CAMP factor (100 µg/ml), GFP (100 µg/ml), or α-toxin (20 µg/ml) in 1% FBS-medium for 18 hr in the presence or absence of desipramine (10 µM). After the incubation, cytotoxicity was measured as described in Experimental Procedures. The data represent mean ± standard error (SE) (n = 8, p<0.05* and p<0.0005*** by Student's t-test, desipramine vs. vehicle control). (0.15 MB JPG) [file pone.0014797.s003.jpg]

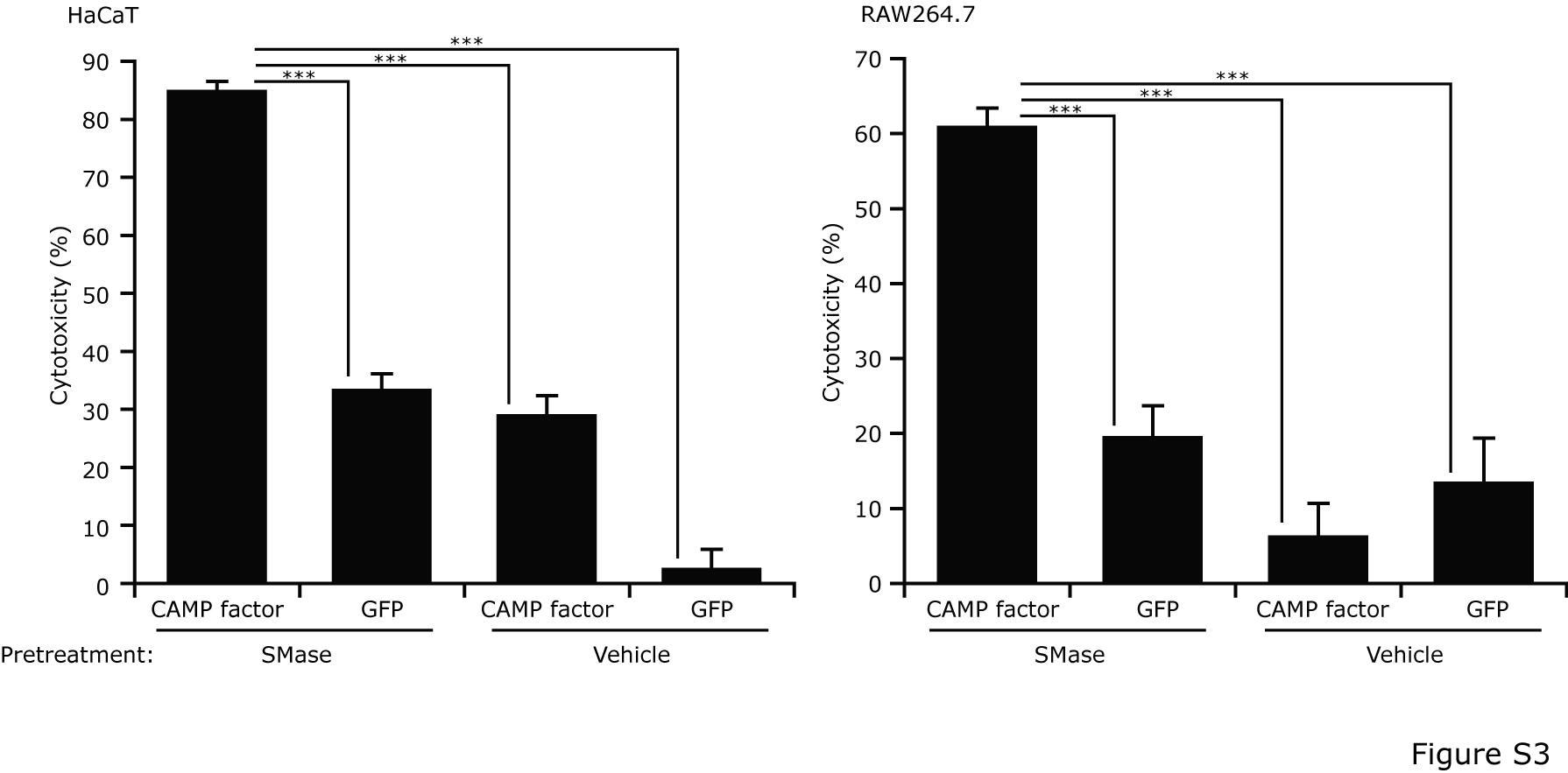

Supplement: Figure S3 — Co-cytotoxic properties of CAMP factor and bacterial SMase in vitro. The HaCaT or RAW264.7 cells were pre-treated with S. aureus SMase (350 mU/ml) or an equal volume of PBS (vehicle) for 15 min and then incubated with 25 µg/ml of recombinant CAMP factor or GFP at 37°C for 18 hr. After the incubation, cell viability expressed as % of cytotoxicity was determined. The data are presented as mean ± SE (n = 6, p<0.0005*** by Student's t-test). (0.18 MB JPG) [file pone.0014797.s004.jpg]
